# Formation Mechanisms of InGaAs Nanowires Produced by a Solid-Source Two-Step Chemical Vapor Deposition

**DOI:** 10.1186/s11671-018-2685-0

**Published:** 2018-08-31

**Authors:** Lei Shang, Longfei Song, Yiqian Wang, Rongsheng Cai, Lei Liu, Fengyun Wang

**Affiliations:** 1 Textile and Clothing Institute, Qingdao University, No. 308 Ningxia Road, Qingdao, 266071 People’s Republic of China; 2 College of Physics and Cultivation Base for State Key Laboratory, Qingdao University, Qingdao, 266071 People’s Republic of China; 3 Nanoscale Physics Research Laboratory, School of Physics and Astronomy, University of Birmingham, Birmingham, B15 2TT UK; 4 School of Materials Science and Engineering, Shandong University of Science and Technology, Qingdao, 266590 China; 5 Key Laboratory of Advanced Energy Materials Chemistry (Ministry of Education), Nankai University, Tianjin, 300071 China; 6 Key Laboratory of Microelectronic Devices and Integrated Technology, Institute of Microelectronics, Chinese Academy of Sciences, Beijing, 100029 China

**Keywords:** InGaAs nanowires, Morphology, Microstructures, Formation mechanism, HRTEM

## Abstract

The morphologies and microstructures of Au-catalyzed InGaAs nanowires (NWs) prepared by a two-step solid-source chemical vapor deposition (CVD) method were systematically investigated using scanning electron microscopy (SEM) and high-resolution transmission electron microscopy (HRTEM). The detailed structural characterization and statistical analysis reveal that two specific morphologies are dominant in InGaAs NWs, a zigzag surface morphology and a smooth surface morphology. The zigzag morphology results from the periodic existence of twining structures, and the smooth morphology results from a lack of twining structures. HRTEM images and energy-dispersive X-ray spectroscopy (EDX) indicate that the catalyst heads have two structures, Au_4_In and AuIn_2_, which produce InGaAs NWs in a cubic phase crystalline form. The growth mechanism of the InGaAs NWs begins with Au nanoparticles melting into small spheres. In atoms are diffused into the Au spheres to form an Au-In alloy. When the concentration of In inside the alloy reaches its saturation point, the In precipitate reacts with Ga and As atoms to form InGaAs at the interface between the catalyst and substrate. Once the InGaAs compound forms, additional precipitation and reactions only occur at the interface of the InGaAs and the catalyst. These results provide a fundamental understanding of the InGaAs NW growth process which is critical to the formation of high-quality InGaAs NWs for various device applications.

**Electronic supplementary material:**

The online version of this article (10.1186/s11671-018-2685-0) contains supplementary material, which is available to authorized users.

## Background

1-D semiconductor nanomaterials such as nanowires, nanotubes, and nanorods have attracted much attention due to their unique properties and the quantum confinement effect [1–14]. In particular, indium arsenide (InAs) nanowires (NWs), due to their confinement effects and high carrier mobility, have been used to extend unique magnetic and electric properties. This makes them a potential candidate for a wide range (880~3500 nm) of optoelectronic device applications and field-effect transistors (FETs) [15–20]. In specific, single InAs NW FETs with an outstanding electron mobility in the range of 3000~10000 cm^2^/Vs have been extensively investigated [15, 18]. However, these devices, based on pristine InAs NWs, always suffer from a large leakage current and small on/off current ratios originating mainly from their small electronic band gap (0.34 eV). Large leakage current and small on/off current ratios affect the switching properties of devices and are of great significance in the practical applications of NW FETs.

Compared with InAs, ternary In_*x*_Ga_1 − *x*_As with tunable chemical stoichiometries and band gap of 0.34 eV ≤ E_g_ ≤ 1.42 eV have been shown to be a good alternative device channel material. This substitution can greatly reduce the leakage current without seriously sacrificing the high electron mobility [19, 21–24]. In our previous works, the relationship between the components of In_*x*_Ga_1 − *x*_As NWs and electrical properties of In_*x*_Ga_1 − *x*_As NWs FETs has been systematically studied. Decreasing the In concentration reduces the off current by about two orders of magnitude and enhances the on/off current ratios by about two orders of magnitude with only a small decrease of mobility [21]. Apart from the composition of NWs, the number of crystal defects, such as twinning planes and stacking faults in III–V NWs, often results from unsuitable growth parameters during the chemical vapor deposition (CVD) method [19, 25, 26–28]. These crystal defects also seriously affect the geometric and the electronic structure. For instance, the zinc blende segments can trap carriers and thereby reduce the electron transport speed in polytypic InP NWs [28]. Hence, it is important to synthesize InGaAs NWs with a controlled structure and defect density to improve their carrier mobility and lifetime in all technological applications. However, at present, due to their complex growth process, there remains considerable challenges to synthesizing In_*x*_Ga_1 − *x*_As NWs (*x* = 0 to 1) with uniform structures and a low defect density along the whole NW. Therefore, designing a growth process to produce high-quality In_*x*_Ga_1 − *x*_As NWs is still quite a challenge [1, 26]. In an attempt to achieve this goal, it is necessary to thoroughly explore the growth mechanism of In_*x*_Ga_1 − *x*_As NWs using the CVD method.

In our previous work, a low-cost, simple two-step growth technique using solid-source CVD was developed to synthesize dense, long, crystalline, and stoichiometric InGaAs NWs with excellent electrical properties. This was done using an amorphous SiO_2_ substrate and Au nanoparticles as catalytic seeds in a vapor-liquid-solid mechanism [19, 21, 22]. It should be noted that these NWs can be parallelly arranged and heterogeneously integrated on various kinds of substrates by a contact printing technique. This demonstrates their promising potential for practical applications when compared with their counterparts grown by the more costly molecular beam epitaxy or metal organic CVD method on crystalline underlying substrates [22, 29]. Although the electrical properties of InGaAs NWs were systematically investigated, the detailed morphology and crystal structures of NWs are insufficiently known [19, 21, 22]. Therefore, the morphology, structural composition, and chemical composition of Au-catalyzed InGaAs NWs grown on amorphous substrates were systematically investigated. The NWs produced in this way have both smooth surfaces and zigzag surfaces. The zigzag surfaces result from the periodic existence of twining structures. At the same time, two kinds of catalyst heads, Au_4_In and AuIn_2_, were also observed in the NWs. Additionally, the orientation relationship between the catalyst head and NW was also studied by HRTEM, and suggested NWs grew following a vapor-liquid-solid (VLS) growth mechanism. The results provide valuable guidance for fabrication of “bottom-up” InGaAs NWs with a smooth surface, minimized twin defects, enhanced crystallinity, and subsequent optimized device performances.

## Methods

### Preparation of InGaAs NWs

**Fig. 1:**
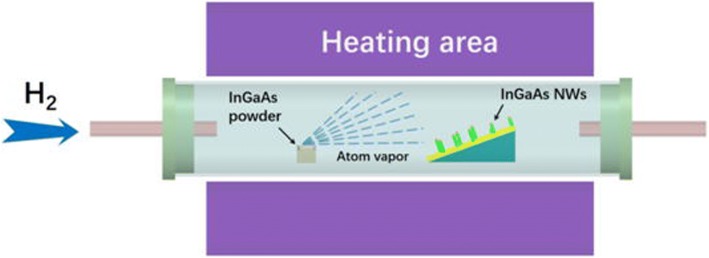
Schematic diagram of the experimental setup for InGaAs NWs

The InGaAs NWs were produced using a solid-source CVD method as previously reported to ensure a high growth yield [15, 16]. The experiment setup is shown in Fig. 1. Briefly, InAs and GaAs powders (99.9999% purity) were mixed together with a weight ratio of 1:1 and put in a boron nitride crucible. This crucible was loaded at the upstream end of the experiment tube to provide Ga, In, and As atoms. The substrate (SiO_2_/Si with the 50-nm-thick thermally grown oxide) with a 0.5-nm-thick Au film to act as a catalyst was loaded in the middle of the downstream zone with a tilt angle of ~ 20°. The distance between the crucible and substrate was held at 10 cm. The substrate was pre-heated to 800 °C and maintained for 10 min in order to obtain Au nanoclusters in the downstream zone, then cooled, and kept at 600 °C. When the downstream zone was at 600 °C, the GaAs and InAs powder zone was heated to 820 °C. The powder zone was held at this temperature for 2 min to allow for the nucleation of the Au catalysts, then the substrate zone was cooled to the growth temperature of 520 °C and maintained for 30 min to be sure the NWs had ample time to grow. Pure H_2_ (99.9995% purity) with a flow rate of 100 sccm was used to carry the evaporated precursors to the substrate and also for purging the system, avoiding NWs being oxidized and obtaining high-quality InGaAs NWs. Subsequently, the source and substrate heaters were switched off, and the system was cooled to room temperature under H_2_.

### Characterization of InGaAs NWs

Morphologies of the grown InGaAs NWs were investigated by SEM (Philips XL30) and bright-field (BF) transmission electron microscopy (TEM, Philips CM-20). Crystal structures of the grown NWs were analyzed by HRTEM-(JEOL 2100F, operating at 200 kV) and the fast Fourier transform (FFT) images. The chemical composition of the grown InGaAs NWs and the catalyst tips were studied by energy-dispersive X-ray (EDX) detector attached to a JEOL 2100F. Selected-area electron diffraction (SAED), bright-field (BF), HRTEM, and EDS examinations were carried out using a JEOL 2100F TEM operating at 200 kV. For the TEM samples, InGaAs NWs were first peeled from the surface of the substrate, dispersed in ethanol by ultrasonication, and dropped onto a holey-carbon-film-coated copper grid.

## Results and Discussion

**Fig. 2:**
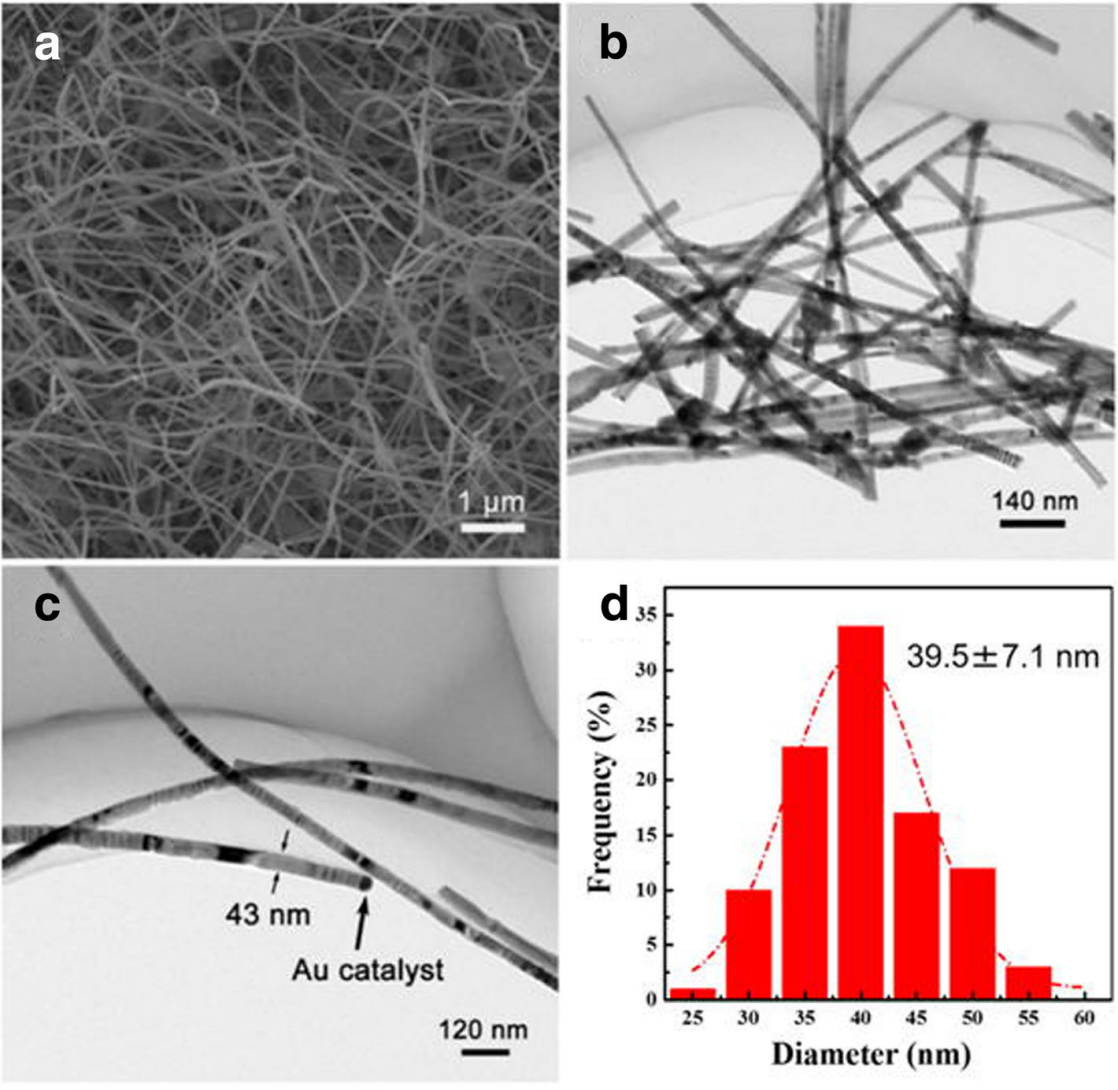
**a** SEM image of the substrate surface after the reaction. **b**, **c** TEM bright-field images of InGaAs NWs. **d** Diameter distribution histogram of InGaAs NWs

As shown in the top-view SEM image of Fig. 2a, the synthesized InGaAs NWs are relatively straight, dense, and longer than 10 μm which is long enough to cross through the narrow channels in FET constructions (< 10 μm). Based on the cross-sectional view SEM images (Additional file 1: Figure S1), the NWs are not perpendicular to the substrate which indicates that no epitaxial growth relationship exists between the substrate and NWs. The BF TEM image (Fig. 2b) also shows InGaAs NWs with a uniform diameter and length. In an effort to determine the diameter distribution of the NWs, 100 NWs were measured. As shown in Fig. 2d, the most common NW diameters are between 30 and 50 nm with an average value of 39.5 ± 7.1 nm. There are only a few NWs having a diameter above 50 nm or below 30 nm. Further investigations using TEM (Fig. 2c) reveal that the InGaAs NW pointed out by the arrows not only has a straight and smooth surface, but also has an obvious Au nanoparticle on its top which implies the InGaAs NWs grown by the VLS growth mechanism are consistent with the previous reports [3, 30]. Other NWs did not exhibit catalytic heads and most of these NWs possess zigzag surface. The catalytic heads may have broken off caused by the twin plan defects during the ultrasonication distribution procedure for TEM grid samples.

**Fig. 3:**
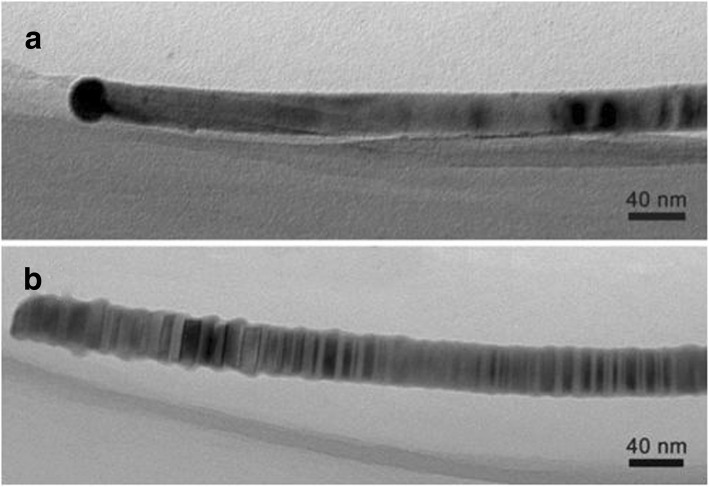
TEM bright-field images of InGaAs NW with two different morphologies, **a** smooth surface and **b** zigzag surface

**Fig. 4:**
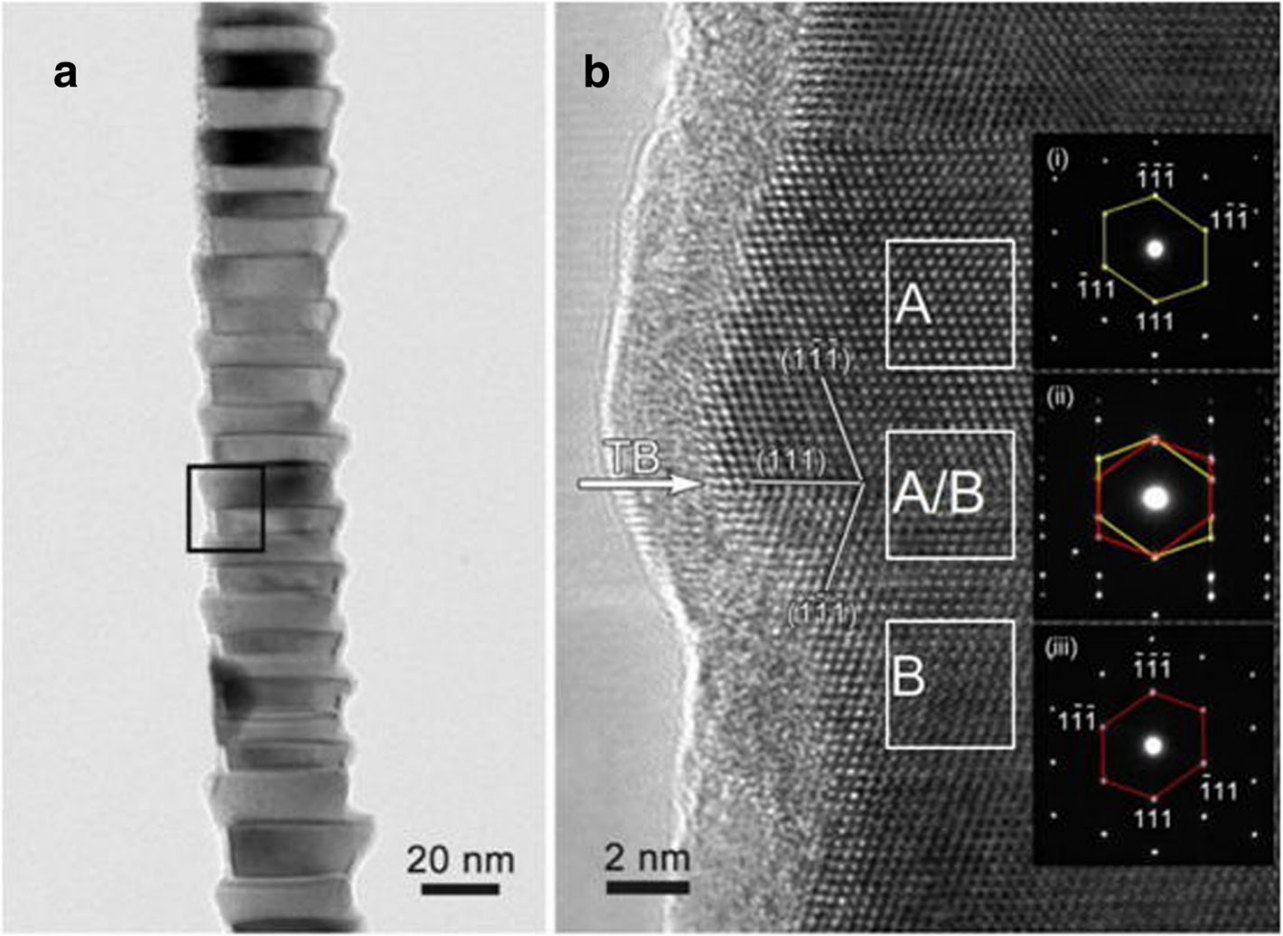
**a** Bright-field TEM image of the zigzag morphology. **b** HRTEM image of the rectangle area marked in **a**, and the insets of (i)–(iii) are the SAED patterns corresponding to the A region, A/B boundary region, and B region, respectively

Based on the highly magnified TEM images (Fig. 3a, b), two distinct morphologies are seen in the InGaAs NWs. Figure 3a demonstrates the NW has a smooth surface and a dark Au catalytic seed. The diameter of both the Au catalyst and NW is ~ 30 nm. Figure 3b shows a NW with a similar diameter (~ 35 nm) to the smooth one, but with a rough surface of many steps, and no catalytic head. In order to investigate the microstructure of these two morphologies, HRTEM was employed. As depicted in Fig. 4a, the BF TEM image clearly demonstrates the NW with the zigzag surface consists of several alternating bright and dark joints appearing in a periodic manner along the NW axial direction, indicating the existence of planar defect structures. Figure 4b is the magnified HRTEM image of the rectangle area marked in Fig. 4a. Based on this magnified HRTEM image, it can be concluded that the zigzag morphology results from the existence of periodic twinning crystals at the twinning boundary marked by the white arrow. The two parts of the twinning crystals share the same (111) crystal plane. The width of the periodic twinning crystals is about 10–20 nm. The insets (i)–(iii) are the SAED patterns taken from region A, the interface of region A/B, and region B, respectively. The insets (i) and (iii) illustrate that the crystals of region A and B all have a cubic zinc blende phase captured along the <110> zone axis of InGaAs, and the growth direction is the <111> direction. The inset (ii) clearly shows that there are two sets of diffraction patterns on the interface of region A/B marked with yellow and red lines. The yellow and red lines show the same diffraction patterns as the insets (i) and (iii), respectively, which further confirms that the zigzag morphology results from the periodic twinning crystals.

**Fig. 5:**
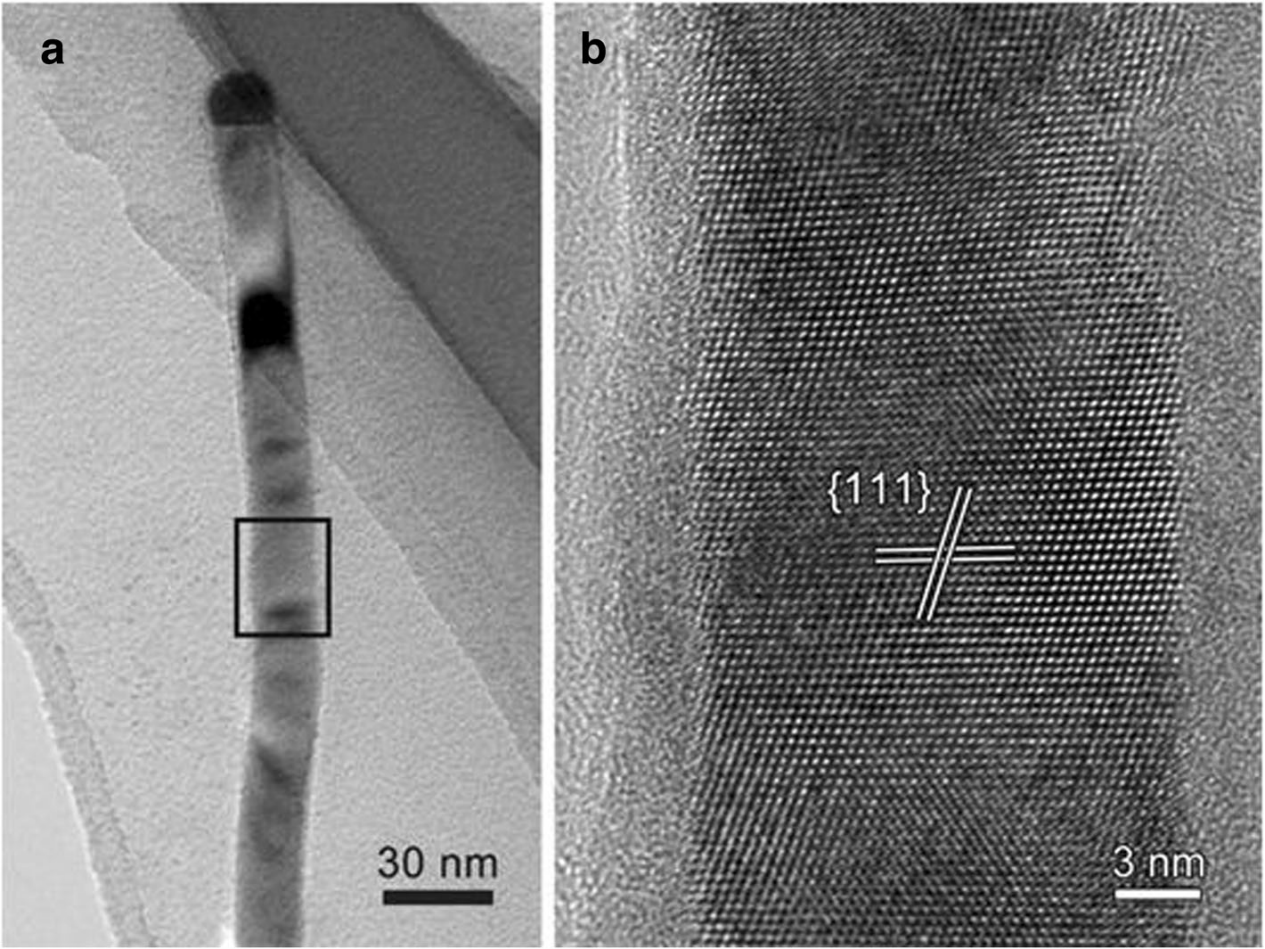
**a** Bright-field TEM image of the NW with a smooth surface. **b** HRTEM image of the rectangle area marked out in **a**

Figure 5a is the HRTEM image of a InGaAs NW which has a smooth surface and no steps or zigzag morphology. Additionally, a semispherical Au nanoparticle located at the end of the NW can be seen, which catalyzes the growth of NWs. In order to compare the microstructure of the smooth NWs with the zigzag ones, an HRTEM image was taken, Fig. 5b, to show the <011> zone axis of the InGaAs. The inter-planar spacing of the crystal planes, marked by two pairs of white lines, measures 3.40 A, which corresponds to the planes in cubic phase InGaAs. According to the systematic investigation of the HRTEM images for more than 40 NWs, it can be concluded that the microstructure of the smooth NWs is different from the zigzag ones. The crystal planes of the smooth NWs are consistent and coherent with few twinning or stacking faults. This indicates that a perfect crystal structure on InGaAs NWs leads to the formation of a smooth surface. More importantly, the smooth surface and the low twin-defect density do not scatter or trap electrons which is beneficial to the carrier transfer along NWs [18, 19]. Twin defects and a rough surface in InGaAs NWs can scatter and trap carriers which gives rise to a serious decline in the electrical performance of NWs [3, 4, 15]. Therefore, it is important to synthesize InGaAs NWs with controllable defect densities and a smooth surface to improve their electrical properties for various technological applications.

**Fig. 6:**
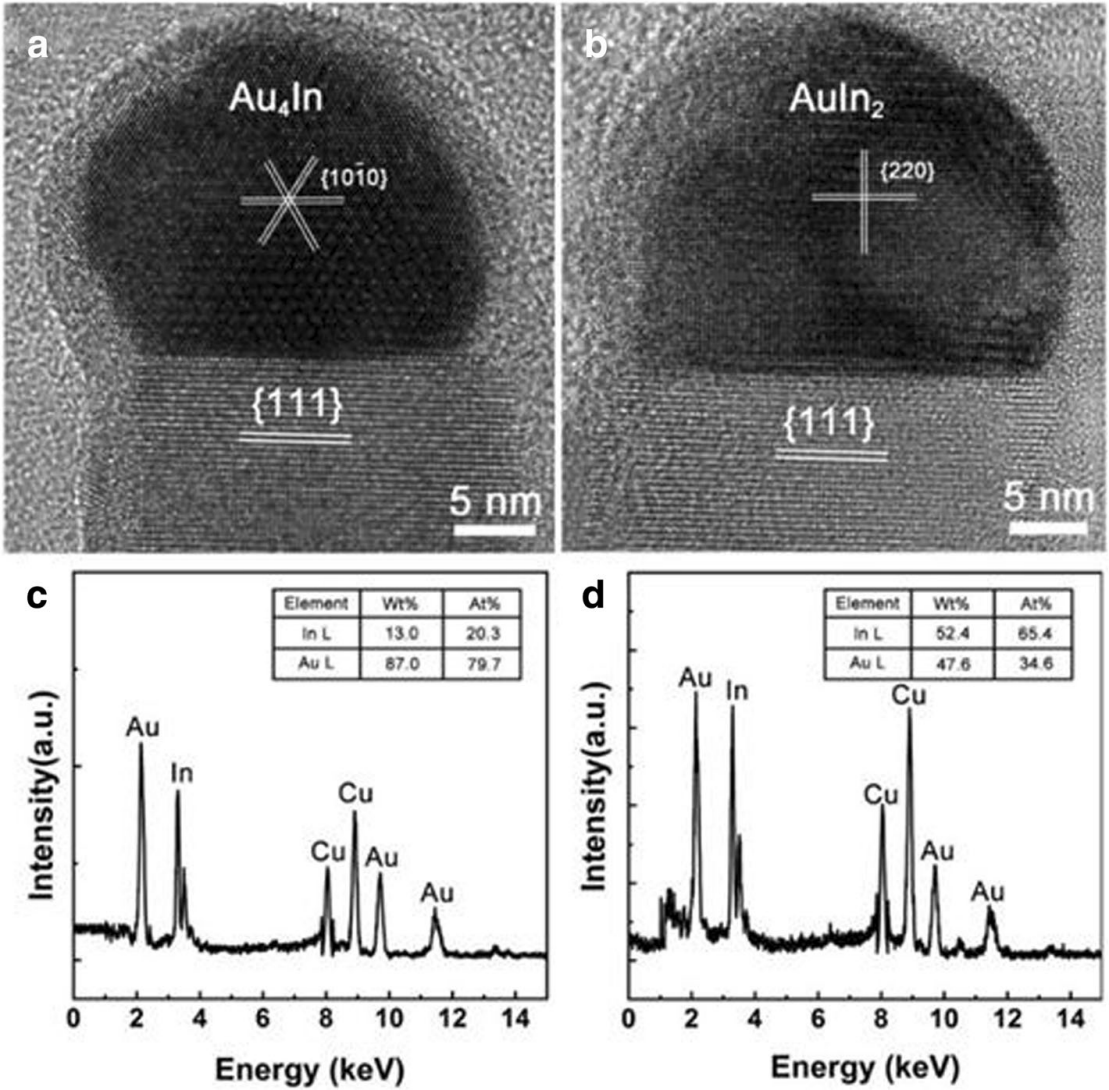
HRTEM images of the Au nanoparticles with two kinds of structures, **a** Au_4_In and **b** AuIn_2_. **c**, **d** EDS of the Au nanoparticles in **a** and **b**, respectively

As reported, the catalyst nanoparticles, especially the composition, microstructure, and orientation relationship with the NWs, play an important role in the formation of NWs produced by CVD method [3]. Therefore, the microstructure of Au nanoparticles on the top of InGaAs NWs was extensively investigated using HRTEM. Based on the compilation of more than 40 NWs, the catalytic heads were mainly found in two forms, Au_4_In with hexagonal structure and AuIn_2_ with cubic structure. Since most NWs with a smooth surface have the same crystalline structure in each trial, the formation of the two kinds of catalyst heads might be caused by slight temperature differences in the cooling speed of the NWs. As depicted in the HRTEM image of Au_4_In nanoparticle (Fig. 6a), the diameter of the catalyst is about 24.8 nm, and this size is similar to the NWs of 23.5 nm. The InGaAs NWs grown along the <111> directions are energetically favorable, and therefore, the NW nuclei aligned with <111> orientation always grow faster and tend to dominate during the growth process (Fig. 6) [3, 13]. The crystal planes marked by the three white lines (Fig. 6a) correspond to the {10-10} planes of Au_4_In which is parallel to the {111} crystal planes of the InGaAs NW with cubic phase structure marked by the two white lines (Fig. 6a). It is explicitly proved that the seed/NW interface orientation relationship is Au_4_In {10-10}|InGaAs {111}. The HRTEM image in Fig. 6b displays another typical cubic-structured InGaAs NW with an AuIn_2_ catalytic head that has a similar diameter of 30.0 nm with that of the NW (30.2 nm). At the same time, the crystal planes marked by the white lines are attributed to {220} planes of AuIn_2_ which is parallel to {111} planes of the prepared NW marked by a parallel pair of white lines (Fig. 6b), indicating the NW grew in the <111> direction. Therefore, it can be concluded that the composition and phase structure of the catalytic heads have no effect on the crystal structure and growth direction of the InGaAs produced by the solid-source CVD method. Both cubic AnIn_2_ and hexagonal Au_4_In can catalyze the growth of InGaAs NWs with uniform cubic phase structure and <111> growth direction, which is beneficial to the large-scale application of InGaAs NWs. To further confirm the composition of the catalyst head, EDX analysis was performed on the catalyst heads shown in Fig. 6a, b, and the corresponding spectra are demonstrated in Fig. 6c, d, respectively. Elements of Cu, Au, and In were detected in the catalysts, but the Cu signal came from the TEM grid and can be ignored completely. The atomic ratios of the Au and In were also quantified to be 4:1 and 1:2, respectively, based on the spectra shown in Fig. 6c, d, which are in agreement with the HRTEM results. It is worth noting that no Ga or As elements were found in the catalyst heads. This may be caused by the low solubility of Ga and As in Au, and thus, Ga and As elements could not diffuse into the catalyst head efficiently [15]. For III–V NWs synthesized by CVD technique, the NW morphology as well as the transport properties are strongly dependent on the components and crystal structure of the catalytic heads. Therefore, the systematic investigation of the Au catalytic head and the relationship between the catalyst and the NWs is critical to understanding the distinctions of carrier transport properties of InGaAs NWs.

**Fig. 7:**
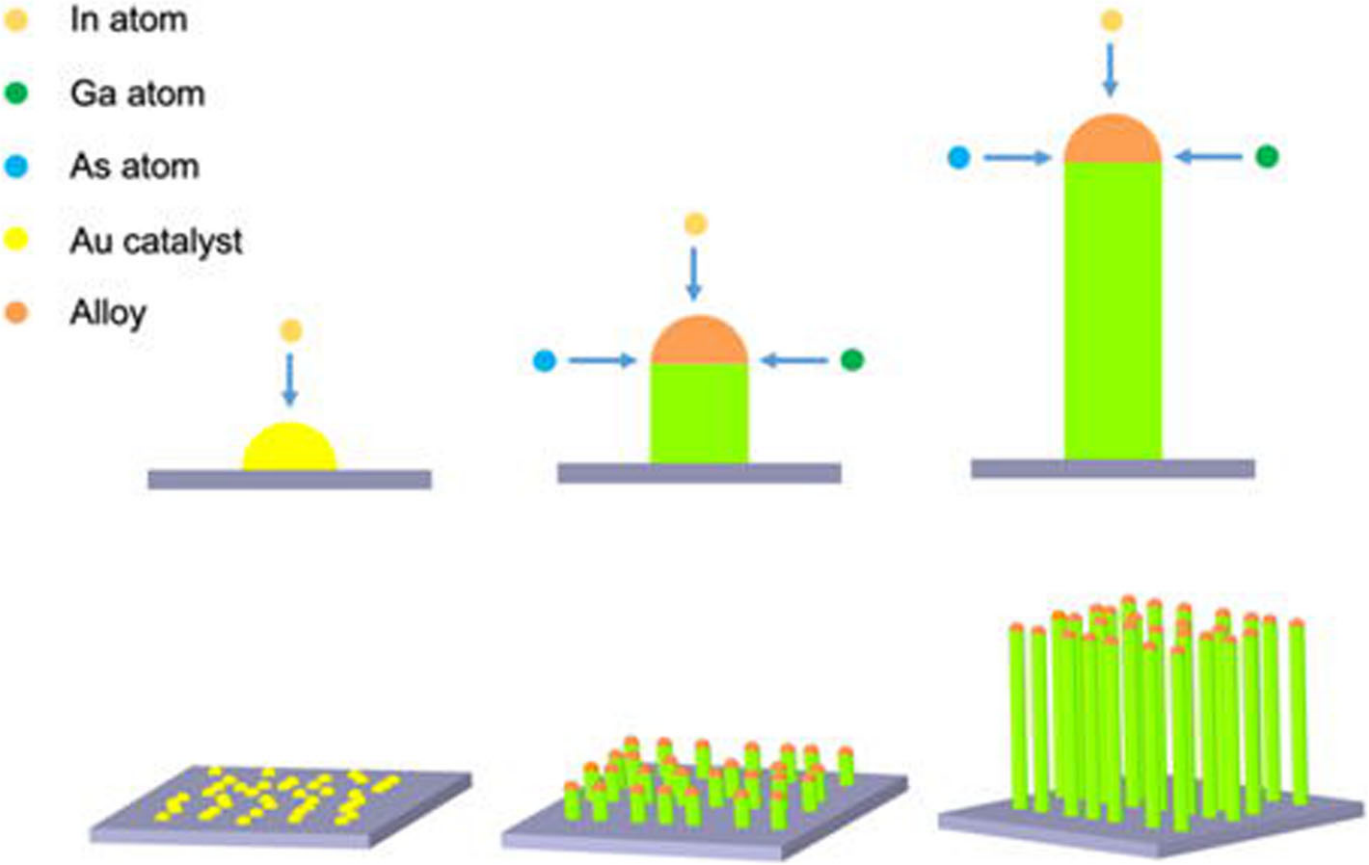
Schematic diagram of the VLS growth mechanism of InGaAs NWs in our studies

Based on the analysis of the HRTEM and EDS results, the VLS growth mechanism of the InGaAs NWs produced by the CVD method can be deduced. Figure 7 is the schematic diagram of the growth process of InGaAs NWs in a tube furnace with a double temperature zone. Firstly, the GaAs and InAs powders were heated at the 820 °C to vaporize Ga, In, and As atoms. Then, this vapor was transported to the substrate for the entire duration of growth under the help of a carrier gas. At the beginning of the reaction, Au nanoparticles were melted into liquid balls on the SiO_2_ substrate at the temperature above the eutectic point of the metal-semiconductor system. Due to the low melting point of the In-Au alloy, the In atoms diffused into the Au nanoparticles and formed the In-Au alloys. Because the solubility of the Ga and As in Au is very low, Ga and As atoms did not diffuse into Au nanoparticles. As the reaction time increased, the concentration of indium became higher and higher, and when In reached its saturation point, In atoms precipitated and combined with Ga and As atoms at the interface of the catalyst and substrate. Once the InGaAs was formed, the precipitation of In with Ga and As only occurred at the interface between the NW and catalyst. Thus, the InGaAs NWs became longer and longer with additional growth time. This growth mechanism is similar to the conventional VLS mechanism [3, 31]. As mentioned before, in this experiment, almost all the NWs grew along the <111> direction mainly because the {111} planes are close-packed and have the lowest energy [3, 21, 22]. Due to the amorphous SiO_2_ growth substrate, the NWs have no specific orientation relationship with the substrate (cross-section SEM images shown in Additional file 1: Figure S1). Moreover, during the formation of NWs, strain forces can form inside the NW due to slight changes in the heating temperature [5, 21]. To release these strain forces, twining structures formed in the NWs which lead to the zigzag morphology. If the strains are fully released, no defects are formed inside the NWs, and smooth parts can also be observed. Moreover, the diameter of the NWs is mainly controlled by the diameter of the catalysts, because the In reacts with Ga and As atoms and precipitates from the catalysts only at the interface between the NWs and the catalysts, which indicates that the fabrication the InGaAs NWs with specific diameters can be produced by adjusting the diameter of catalyst.

## Conclusions

In conclusion, InGaAs NWs can be successfully synthesized by the CVD method. The average diameter of the NWs was 39.5 ± 7.1 nm, and their growth direction was <111>. The NWs displayed two surface morphologies, a zigzag surface and a smooth surface. Their appearances are random and can also occur in the same NW. HRTEM investigation reveals that the zigzag morphology results from the periodical existence of twining structure which is mainly due to the strain forces inside the NW. The formation mechanism of the NWs begins with the Au nanoparticles being melted into small balls and the In atoms being diffused into the Au ball to form an Au-In alloy. When the In concentration reaches its saturation point, In atoms precipitate and combine with Ga atoms and As atoms at the interface between the catalyst and substrate, forming InGaAs. The precipitation of the InGaAs only occurs on the interface of the InGaAs and the catalyst. With increased reaction times, long InGaAs NWs formed on the substrate. Additionally, the diameter of the NWs seemed to be determined by the size of catalysts. The two catalysts, Au_4_In and AuIn_2_, both produce cubic-structured InGaAs NWs with <111> growth direction. All findings give further understanding of the synthesis of high-quality InGaAs NWs with optimized device performance for future technical applications.

## Additional File

Additional file 1: **Figure S1.** The cross-section SEM images of the InGaAs NWs. (DOC 1615 kb)

## Abbreviations

EDX: Energy-dispersive X-ray spectroscopy
HRTEM: High-resolution transmission electronic microscopy
NWs: Nanowires
SAED: Selected-area electron diffraction
SEM: Scanning electron microscopy
TBs: Twin boundaries
TEM: Transmission electronic microscopy
