# Supplementary material for: Formation Mechanisms of InGaAs Nanowires Produced by a Solid-Source Two-Step Chemical Vapor Deposition
Source: Nanoscale Res Lett. 2018 Aug 31;13:263. doi: 10.1186/s11671-018-2685-0 (PMC6119172; doi:10.1186/s11671-018-2685-0)
Supplement: Supplementary file 1 — Figure S1. The cross-section SEM images of the InGaAs NWs. (DOC 1615 kb) [file 11671_2018_2685_MOESM1_ESM.doc]

**Supporting Information**

**Formation Mechanisms of InGaAs Nanowires Produced by a Solid Source Two-Step Chemical Vapor Deposition**

**Lei Shang^1^, Longfei Song^2^, Yiqian Wang^2^, Rongsheng Cai^3^, Lei Liu^4,5^, Fengyun Wang^2,6^**

1. Textile & Clothing Institute, Qingdao University, No. 308 Ningxia Road, Qingdao 266071, P.R China.

2. College of Physics and Cultivation Base for State Key Laboratory, Qingdao University, Qingdao 266071, P.R China.

3. Nanoscale Physics Research Laboratory, School of Physics and Astronomy, University of Birmingham, Birmingham B15 2TT, United Kingdom.

4. School of Materials Science and Engineering, Shandong University of Science and Technology, Qingdao 266590, China.

5. Key Laboratory of Advanced Energy Materials Chemistry (Ministry of Education), Nankai University, Tianjin 300071, China.

6. Key Laboratory of Microelectronic Devices & Integrated Technology, Institute of Microelectronics, Chinese Academy of Sciences, 100029, China.


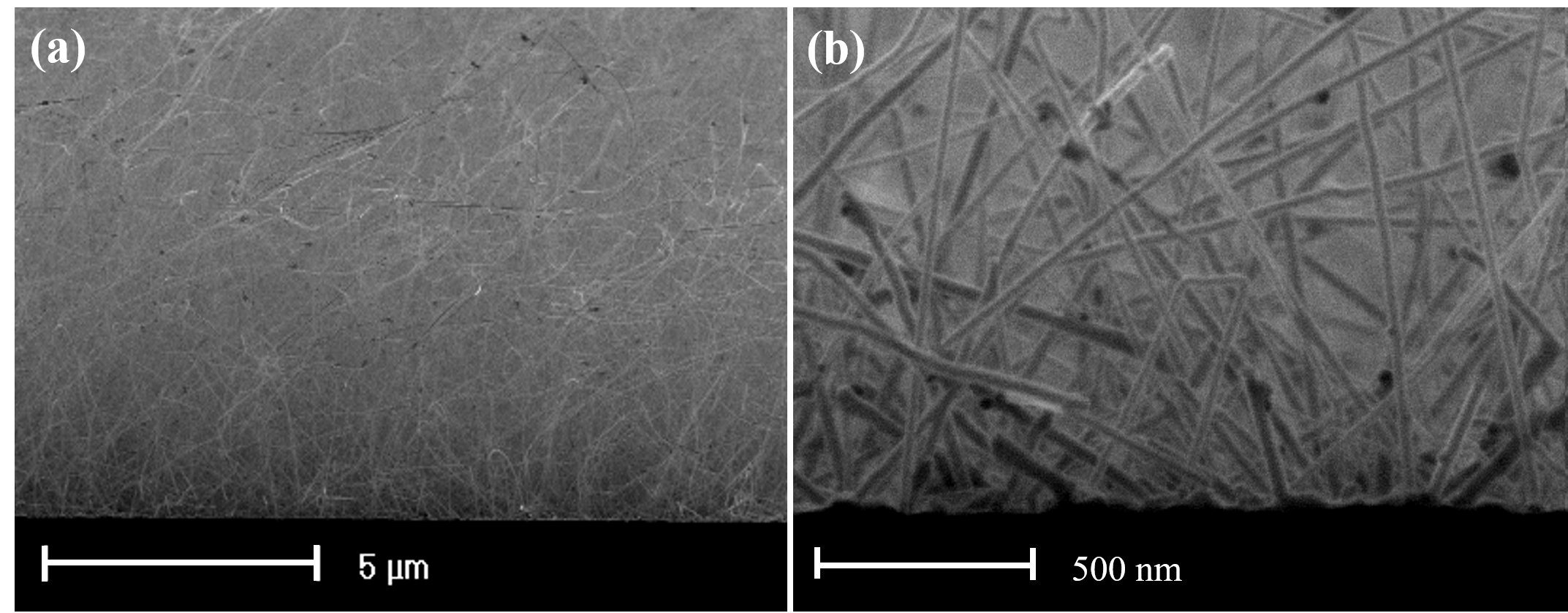


Figure S1. The cross-section SEM images of the InGaAs NWs.
